# 10th Anniversary of a Two-Stage Design in Bioequivalence. Why Has it Still Not Been Implemented?

**DOI:** 10.1007/s11095-020-02871-3

**Published:** 2020-07-13

**Authors:** Michał Kaza, Alexander Sokolovskyi, Piotr J. Rudzki

**Affiliations:** 1 Pharmacokinetics Department, Łukasiewicz Research Network - Pharmaceutical Research Institute, 8 Rydygiera Str., 01-793 Warsaw, Poland; 2 Farmak JSC, Clinical Trial Department, 63 Kyrylivska Street, Kyiv, Ukraine

**Keywords:** Two-stage design, sequential design, bioequivalence, pharmacokinetics, in vitro dissolution

## Abstract

**Purpose:**

In 2010 the European Medicines Agency allowed a two-stage design in bioequivalence studies. However, in the public domain there are mainly articles describing the theoretical and statistical base for the application of the two-stage design. One of the reasons seems to be the lack of practical guidance for the Sponsors on when and how the two-stage design can be beneficial in bioequivalence studies.

**Methods:**

Different variants with positive and negative outcomes have been evaluated, including a pivotal study, pilot + pivotal study and two-stage study. The scientific perspective on the two-stage bioequivalence study has been confronted with the industrial one.

**Results:**

Key information needed to conduct a bioequivalence study – such as in vitro data and pharmacokinetics – have been listed and organized into a decision scheme. Advantages and disadvantages of the two-stage design have been summarized.

**Conclusion:**

The use of the two-stage design in bioequivalence studies seems to be a beneficial alternative to the 2 × 2 crossover study. Basic information on the properties of the active substance and the characteristics of the drug form are needed to make an initial decision to carry out the two-stage study.

## Introduction

We have had more than 2200 results of bioequivalence studies in the last 32 years [1]. Since 2010, the European Medicines Agency (EMA) has allowed a two-stage design in bioequivalence studies [2]. A two-stage design is one of the alternative designs, group-sequential and adaptive designs, that have been considered for clinical use since the 1970s [3]. The alternative approach to a clinical trial design, accepted by FDA [4], EMA [5] and Health Canada [6], is widely used in innovative research due to significantly higher savings and no unnecessary exposure of people to drugs. It seems that this approach may also be convenient for bioequivalence studies. In accordance with the EMA guideline [2], the number of participants can be expanded if bioequivalence has not been demonstrated in the first group of subjects. The results for the initial and the second group are combined for the final assessment. However, the decision to conduct the two-stage clinical trial must be made before the study begins. And this requires an answer to the following question: when the two-stage model is better than the standard cross-over design.

There are actually more articles describing the theoretical and statistical base for the application of the two-stage design [3, 7–25] than there are reported studies – only two have been reported since 2010 [1]. There are also few papers describing two-stage bioequivalence studies [26, 27]. Admittedly, proposals for the use of the two-stage procedure in bioequivalence studies appeared in the 80s [28], the papers of Potvin et al. [7] and then of Montague et al. [8] became the starting point for consideration of the use of the two-stage design in bioequivalence studies. Subsequent authors focused on controlling type I error, selection of the optimal number of subjects (and its impact on study power), defining stopping rules and modifying previously described models [3, 9–25]. A lot has been said, explained, simulated, checked and proven. As a result, we know how to perform a statistical evaluation, don’t we? So why has so few bioequivalence studies been done as a two-stage design? Why do Sponsors – usually generic drug companies - avoid this type of study design?

One of the reasons might be scepticism of regulatory authorities regarding simulation-based methods resulting in caution of companies in proposing such a model. While FDA [4] and Health Canada [6] recommended the simulation based on two-stage designs European regulatory acceptance is limited [5]. Another reason seems to be the lack of practical guidance for Sponsors on when and how the two-stage design can be beneficial in bioequivalence studies. Advanced statistical simulations and arguments may give the impression that the two-stage study is complicated and risky. Available papers focus on the intricacies of statistical analysis, treating the advantages and disadvantages of the two-stage design at random. There is a lack of tools that would help the pharmaceutical industry to decide if the two-stage design is a good option for a particular study. This paper aims to fill this niche.

## Materials and Methods

To evaluate a number of bioequivalence studies Clinicaltrial.gov, Pubmed and Web of Science databases have been searched using the following keywords: “adaptive design”;” two-stage design”;” bioequivalence”.

From the beginning our study on two-stage bioequivalence has incorporated both the scientific and the industrial perspective. Different variants with positive and negative outcomes have been evaluated, including: a pivotal study, pilot + pivotal study and two-stage pivotal study. Key information needed to conduct a bioequivalence study – such as in vitro data and pharmacokinetics – have been listed and organized into a decision scheme. Advantages and disadvantages of the two-stage design have been summarized.

The two-stage design in this paper is based on adaptive sequential sample size Method B by Montague et al. 2012 [8]. In this case, the Sponsor is “punished” for choosing the two-stage design and alpha = 0.0294 is used to assess bioequivalence after the first stage of the study., i.e. type I error rate (patient’s risk) should not exceed 2.94%.

In our work we define the two-stage design study as a two-stage, two-period cross-over study. We use the terms *small / medium/ large group of subjects*, *short / long half-life of drug* without defining specific numerical ranges. The number of participants in the bioequivalence study must not be less than 12 [2]. One can consider a group of over 30–40 participants to be large, but this value may change depending on the active substance and the Sponsor’s approach. On the other hand, the half-life is considered to be short if its value is given in hours (not in days).

## Results

**Fig. 1:**
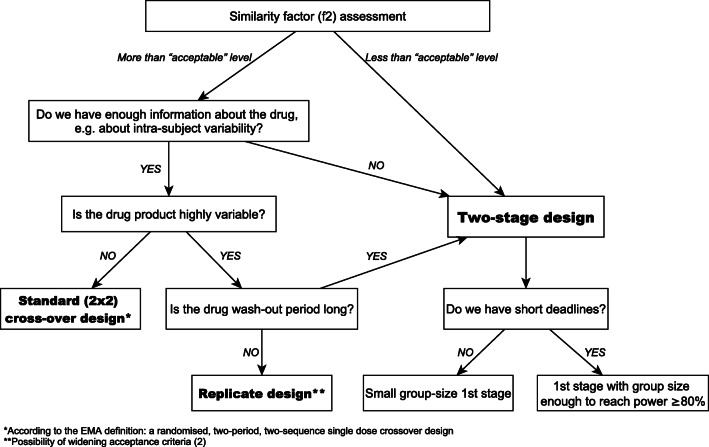
The decision scheme in bioequivalence study

To estimate the sample size for a standard cross-over design one needs to know intra-subject variability and test/reference ratio (T/R), also called the geometric mean ratio (GMR) of pharmacokinetic metrics [3, 25]. For a given drug form both are known only after the bioequivalence study. However, knowledge about the first parameter is more or less available in the public domain in the form of Public Assessment Reports and publications, whereas T/R can only be estimated from in vitro tests prior to the study. Therefore, let’s forget about this parameter for a moment because is not needed as such it when making decisions on the bioequivalence study in accordance with our decision scheme (Fig. 1). Let’s start from the moment when we assess the release profiles of a new drug candidate.

Dissolution profile similarity testing is a step of choice. The requirements for similarity of dissolution profiles are defined by a similarity factor (f2). Its value between 50 and 100 suggests that the two dissolution profiles are similar [2]. Under the assumption that the in vivo profiles will reflect the kinetics of drug measured in vitro, the following considerations can be made. In fact, meeting the acceptance criterion at the acceptable level 50 is not a good premise for starting the clinical part of the study. The best option is the f2 value over 80, but the result within 70–80 can also be considered good. The f2 value below 70 increases the risk of the bioequivalence study failure. If the uncertainty about the form of the drug is large, one can consider the two-stage design. The study can be started with a small or large enough group of participants – minimum 12 subjects is required for any clinical study [2].

Depending on the project, we have different amounts and quality of pharmacokinetic data. Key information in the study planning process is intra-subject variability. If comprehensive drug information does not indicate a high intra-subject variability, the standard crossover design is the study of choice. According to the EMA definition it means a randomized, two-period, two-sequence single dose crossover study [2]. Parallel design may be useful when studying drugs with very long half-life. In the case of highly variable drugs, attention should also be paid to the half-life of the drug. For highly variable drugs with a short half-life (values in hours, not days), the best solution seems to be a replicate design [29]. In another case the study can be carried out using the two-stage design in the simplest model of two-period cross-over.

## Discussion

The requirements for similarity of dissolution profiles are defined by a similarity factor (f2). One should remember that dissolution similarity (f2) or another method is informative only for BCS class I and - under certain circumstances – for class III drugs [9]. Taking f2 factor into account when planning the bioequivalence study allows us to get as much information as possible about the drug products studied. However, the decision to start a clinical trial also depends on other factors related to the study: clinical trial scheme (including its duration), stability of the drug form, corporate deadlines, risk of failure, costs of the study, etc. Therefore, the “level of acceptance” differs in different projects. Regardless of this level, lower f2 value indicating differences between drug products may be a clue to consider the two-stage design [3].

The older the substance, the more data is missing and the more difficult it is to plan a bioequivalence study. Lack of Public Assessment Reports may mean a lack of information on intra-subject variability and on maximum concentration for a given drug dose or form. In this case, the two-stage design may be an alternative to the pilot study. The advantage of the two-stage model over both the pilot and pivotal study is that only one agency’s approval is needed to conduct such bioequivalence study. The study can be started with a small group of participants which means a lower cost of the study and a shorter implementation time. Then it can be continued or stopped. This guarantees the safety of the participants and protects the Sponsor from investing in a product that is not bioequivalent. When reducing financial risk is the main goal, then the small size at 1st stage is the optimal solution. However, it should be noted that opinions about small initial groups are divided. The prevailing belief is that it is not a good idea to have a low size of initial group [9].

Key information in the study planning process is intra-subject variability. Without it, one cannot predict the number of study participants properly. In turn, the wrong number of study participants affects the cost of the study or results in the insufficient statistical power (below 80%). This also applies to incomplete data or data related to different forms, e.g. oral suspension vs. tablets. Incomplete data as well as high intra-subject variability increase the Sponsor’s risk. Using the two-stage design appears to be the solution in this case [16, 20, 22, 23]. Simulations by Fenta et al. [13] indicate the need for caution when two factors overlap, high drug variability and deviations between test and reference medicinal products. This may result in a disproportionately large group of participants for the second stage of the study. In this case, the replicate study is the recommended alternative.

For highly variable drugs a replicate design is a step of choice. However, the replicate study is extended in time because – depending on the model chosen – it consists of at least three periods. Further extension of the study due to a long half-time / long wash-out period increases the risk of participant drop-out. And since there is already an increased risk in such a study due to the high drug variability, it would be great to reduce it. Theoretically, this can be done by using the two-stage model: starting the study with a large enough group of participants – sufficient to reach the power of ≥80%. However, widening the acceptance range for C_max_ based on intra-subject variability of the reference drug (2) might be problematic in this case. The computational methods proposed by EMA may lead to an increase of type I error [16, 20] and in the worst-case exceeding the required patient’s risk. Therefore, two-stage model for highly variable drugs, if any, should be applied with extreme caution.

A limitation of our paper is that we did not consider conducting a parallel study in a two-stage model. Parallel design is used relatively rarely in bioequivalence studies, so there are not many computational methods described [12]. Still it is an important area for future research as it may help property design studies for very long half-life drugs and drugs inducing or inhibiting its own metabolism.

To conclude, the use of the two-stage design in bioequivalence studies seems to be a beneficial alternative to the 2 × 2 crossover study (Table 1). Basic information on the properties of the active substance and the characteristics of the drug form are needed to make an initial decision to carry out the two-stage study. Statistical methodology for two-stage studies to assess bioequivalence is sufficiently developed and described. However, further research is needed on stopping rules / stopping criteria [3, 14] which are recommended by the EMA guideline but not defined. We hope that this paper will convince Sponsors about the advantages of the two-stage design in particular cases. Wider use of the two-stage design may be beneficial to study participants and pharmaceutical companies. We hope that this paper will inspire further research (including simulations) and discussion of this topic.

**Table 1:** Different designs of bioequivalence studies – a comparison of characteristics

Type of study	Standard*	2-stage	Pilot + Pivotal
Regulatory approval only once	+	+	may be possible
Protection against unpredictable variability		+	+
Protection against investing in a medicinal product that is not bioequivalent		+	+
Group size	small	medium	large
Duration	short	medium	long

*According to the EMA definition: a randomised, two-period, two-sequence single dose crossover design
